# Transcriptome-wide analysis reveals the progress of *Cordyceps militaris* subculture degeneration

**DOI:** 10.1371/journal.pone.0186279

**Published:** 2017-10-26

**Authors:** Juan Yin, Xiangdong Xin, Yujie Weng, Zhongzheng Gui

**Affiliations:** 1 School of Biotechnology, Jiangsu University of Science and Technology, Zhenjiang, Jiangsu, China; 2 Sericultural Research Institute, Chinese Academy of Agricultural Science, Zhenjiang, Jiangsu, China; Institute of Plant Physiology and Ecology Shanghai Institutes for Biological Sciences, CHINA

## Abstract

**Background:**

The entomopathogenic mushroom *Cordyceps militari*s is an important medicinal and food resource owing to its various medicinal components and pharmacological effects. However, the high frequency of strain degeneration during subculture seriously restricts the large-scale production of *C*. *militari*s, and the mechanism underlying strain degeneration remains unclear. In this study, we artificially cultured *C*. *militaris* for six generations and compared changes during fruiting body growth. The transcriptome of six generations of *C*. *militaris* strains were sequenced with the Illumine Hiseq4000.

**Results:**

The subcultured *C*. *militari*s strains degenerated beginning at the third generation, with incomplete fruiting body growth beginning at the fourth generation. Over 9,015 unigenes and 731 new genes were identified. In addition, 35,323 alternative splicing (AS) events were detected in all samples, and more AS events occurred in the second, fourth and sixth generations. Compared with the first generation, the third generation (degenerated strain) included 2,498 differentially expressed genes (DEGs) including 1,729 up-regulated and 769 down-regulated genes. This number was higher than the number of DEGs in the second (1,892 DEGs), fourth (2,006 DEGs), fifth (2,273 DEGs) and sixth (2,188 DEGs) generations. Validation of RNA-seq by qRT-PCR showed that the expression patterns of 51 DEGs were in accordance with the transcriptome data.

**Conclusion:**

Our results suggest that the mechanism of *C*. *militaris* strain degeneration is associated with gene involved in toxin biosynthesis, energy metabolism, and DNA methylation and chromosome remodeling.

## Introduction

*Cordyceps militaris*, a tonic herb used in traditional Chinese medicine, is an economically valuable, edible entomopathogenic mushroom that is used clinically as a substitute of *Cordyceps sinensis* owing to its similar chemical components and pharmacological activities [1]. *C*. *militaris* contains several pharmacologically active ingredients, such as cordycepin, adenosine, polysaccharide, ergosterol, that exhibit significant biological activities known to protect and improve lung and kidney function [2], and to immunomodulatory [1,3], antioxidant [4], anti-tumor [5,6,7],anti-inflammatory [8], and hypotensive effects [9].

Because naturally occurring *C*. *militaris* is rare, cultivated *C*. *militaris* fruiting bodies are currently commercially available as medicinal materials and health food products in China, Korea, and Southeast Asia [10]. This artificial cultivation has improved the imbalance between the supply and the demand of wild *C*. *militari*s [11]. However, the high frequency of strain degeneration during subculture and preservationof *C*. *militari*s has limited its large-scale production and industrial process [12]. Strain degeneration results in the significant fluctuations in sporulation quantity, deficiencies in fruiting body formation, reductions in secondary metabolite yields, and other irreversible hereditary phenotypes, leadings to considerable economic losses [13].The strain degeneration of *C*. *militaris* involves complex regulatory processes and involves many factors [14]. Studies have shown that mating type change, DNA methylation, genetic mutations, harmful substance accumulation, and virus infections are all precipitating factors of *C*. *militaris* degeneration [15].

The genome sequence of *C*. *militaris* and the transcriptomes of the mycelium and fruiting body have been analyzed by next-generation sequencing (NGS) [16,17]. However, the molecular mechanism of strain degeneration remains unclear. In this study, we cultured *C*. *militaris* for six generations and compared changes in fruiting body growth during subculture. We also analyzed the transcriptomes of different generations of *C*. *militaris* using Illumine Hiseq4000 technology. Subsequently, the differentially expressed genes (DEGs) as determined by RNA-Seq were analyzed using Gene Ontology (GO) and Kyoto Encyclopedia of Genes and Genomes (KEGG) databases and were validated by quantitative real-time PCR (qRT-PCR). Our results provide a better understanding the transcriptomic changes that occur in *C*. *militari*s during subculture and the underlying molecular mechanism of strain degeneration.

## Materials and methods

### Strain and sample preparation

Wild-type of *C*. *militaris*, designated YCC, was collected from Maoshan hilly area (at altitude 267.5 m) without specific permissions, Jiangsu province, China and isolated by tissue separation in the asexual phase by the Laboratory of Hi-Tech Processing of Sericultural Resources, Sericultural Research Institute, Chinese Academy of Agricultural Sciences, China.

For the collection of different generations following asexual development, the strain was inoculated onto potato dextrose agar (PDA) plates (20% potato, 2% dextrose, 1.5% agar and 1% peptone, w/v) and incubated at 23°C on a shaker at 150 rpm for 7 d in the dark. Mycelia were inoculated into rice medium, cultivated in the dark at 23°C for 10 d, and then kept at 23°C under a 17:7 h dark/light cycle for fruiting body production [18]. The mycelia and fruiting body of this strain were subcultured for the acquisition of second, third, fourth, fifth, and sixth generations, designated YCCZ2, YCCZ3, YCCZ4, YCCZ5, and YCCZ6, respectively. Mycelium from all six generations were collected for DNA and RNA extraction.

### RNA extraction and sequencing

Total RNA from each sample was extracted using RNeasy Plant Mini kit (Qiagen Co. LtD., Beijing, China) and the residual genomic DNA was digested using RNase-free DNase I according to the manufacturer’s protocol. Poly (A) mRNA was enriched using oligo (dT) beads and fragmented using fragmentation buffer. Finally, 100 ng purified and enriched mRNA was used to construct a cDNA library for each sample using NEBNext® Ultra^TM^ RNA Library Prep Kit for Illumina (New England Biolabs, Ipswich, MA, USA). cDNA fragments of 200 bps (± 25 bps) were selected and purified by gel-electrophoresis and used as templates for amplification with PCR for end- repair and poly (A) addition. Purified library products were evaluated using the Agilent 2200 Tape Station and Qubit 2.0 software (Life Technologies, Carlsbad, CA, USA). RNA-Seq was performed at Gene Denovo Co., Ltd. (Shenzhen, China) using the HiSeq TM 4000 (Illumina, San Diego, CA, USA).

### Transcriptome assembly and function annotation

Prior to sequencing, raw data were filtered to produce high-quality clean data. Clean reads were then assembled de novo into longer contigs based on overlapping regions using the Trinity platform (http://trinityrnaseq.sourceforge.net/) [19]. All subsequent analyses were performed using clean data. For annotation, clean data were mapped to the *C*. *militaris* genomic data (BioProject acession no. PRJNA225510) from the NCBI transcriptome reference database.

All splice junction sites of the same gene (≥ 5 reads) were determined and compared with the reference splice junction sites (≤ 1 base) using TopHat align to distinguish new alternative splicing (AS) events. DEGs were filtered and identified according to the edgeR criteria (http://www.Bioconductor.org/packages/release/bioc/html/edgeR.html). Functional annotation (GO terms) were downloaded from Uniprot database (http://www.uniprot.org/uniprot). For GO enrichment analysis, GO annotations for each DEG were retrieved by mapping to GO terms in the database at http://www.geneontology.org. For KEGG pathway analysis, KEGG orthology terms for DEGs were retrieved from the KEGG pathway database (http://www.genome.jp/kegg/). Cluster analysis of gene expression patterns was performed using cluster software [20] and Java Treeview software [21]. All expressed genes in the current transcriptomes were annotated based on BLAST homology searches and searched against the Swiss-Prot and TrEMBL databases by double-direction BLAST. To further explore the function of DEGs in different samples, KEGG enrichment analyses were performed using hypergeometric tests with the Blastall software.

### Validation of RNA-Seq by quantitative real-time PCR

qRT-PCR was carried out to validate the quality of RNA-Seq data. Total RNA was extracted as described above. Each RNA sample was treated with RNase-free DNase I (TaKaRa, Shiga, Japan) following the manufacturer's protocol to remove any residual genomic DNA. DNase I-treated RNA (2 mg) was subjected to reverse transcriptase using oligo (dT) primer and PrimeScript^TM^ Reverse Transcriptase (TaKaRa, Shiga, Japan) according to the manufacturer's protocol. Total RNA (2 μg) was used to synthesize cDNA with PrimeScript™ RT reagent Kit (Perfect Real Time, TaKaRa, Japan). The *C*. *militaris* housekeeping gene, glyceraldehyde-phosphate dehydrogenase (*Cmgapdh*), was used as an internal control for normalization. Primers for the qRT-PCR of 51 DEGs were designed with Premier 6.0 software and are shown in S1 Table. Three biological replicates were performed per sample.

### Data analysis

Sequence similarity was analyzed using Blast software. Multiple sequence alignment analysis was carried out with Clustalx W software. All experiments were repeated at least three times, and the results are expressed as mean ± S.D. Statistical evaluation was done using ANOVA (SPSS 18.0), with p < 0.05 considered significant.

## Results

### Changes in the morphological characteristics of the *C*. *militaris* fruiting body in different generations

**Fig 1:**
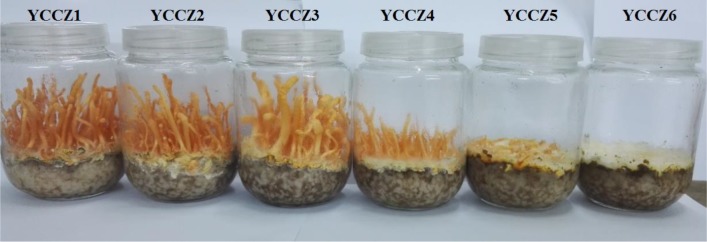
Changes in the *C*. *militaris* fruiting body at different generations.

The growth of *C*. *militaris* fruiting body is an important morphological characteristics in evaluating strain degeneration during subculture. *C*. *militaris* was subcultured in rice medium for six generations, and changes in morphological characteristics of fruiting body were observed after 40 days of cultivation (Fig 1). In the third generation, the fruiting body became stronger and shorter, while the size of fruiting body was significantly reduced in the fourth generation. The strain appeared to be completely degenerated in the fifth generation, and only mycelia grew in the sixth generation. As shown in Fig 1, the subculture of *C*. *militaris* induced strain degeneration beginning in the third generation.

### Transcriptome sequencing and assembly

As shown in Table 1, the average number of clean reads per sample was 53,118,184. After quality filtering, over 6×10^9^ bps of clean data was obtained across the different samples (S2 Table), and more than 98% HQ clean reads were identified (S1 Fig). About 90% reads were mapped to the *C*. *militaris* genome sequence (S3 Table). In total, 9,651 genes were detected in all transcriptomes, including 9,015 known genes and 731 novel genes. The number of new gene in each generation from YCCZ1 to YCCZ6 was 668, 705, 699, 704, 702 and 699, respectively (Table 1). The six samples showed similar matching results, with ~90% of reads matching the predicted genes in the genome and ~70% being unique matches (S2 Fig). The above results revealed that our sample data were reasonable with good correlations among biological replicates and that sequencing data could be used for following analyses.

**Table 1:** Summary of sequencing data utilized in this study.

Sample	Clean Reads	HQ Clean reads	Clean Base	Total mapped	Total mapped Ratio (%)	Unique Match(%)	Known Gene	New Gene	All Gene
**YCCZ1**	41009930	40623584	6062919977	11603568	70.23	70.12	8586 (88.96%)	668	9254
**YCCZ2**	59291792	58701420	8755948868	11607702	70.30	70.17	8735 (90.51%)	705	9440
**YCCZ3**	47879596	47274196	7052765933	14351858	69.17	69.04	8714 (90.29%)	699	9413
**YCCZ4**	56986574	56336928	8406700715	16735019	69.68	69.54	8745 (90.61%)	704	9449
**YCCZ5**	52911126	52298678	7800954755	15641003	69.90	69.77	8700 (90.15%)	702	9402
**YCCZ6**	60630086	60093188	8971315366	17870079	69.05	68.89	8746 (90.62%)	699	9445

Note: Clean Reads = Raw Reads—Adapter—Low Quality–High N rate

HQ Clean Reads = Raw Reads—Clean Reads

Known Gene Percentage = number of detected / total genes in reference genome.

### Analyses of differentially expressed genes (DEGs)

**Fig 2:**
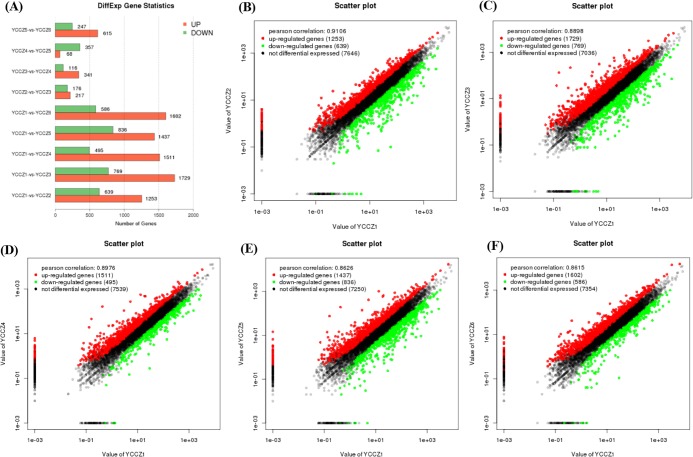
Differentially expressed genes (DEGs) analyses. Note: (A) DEGs statistics of different samples. (B) Scatter plot of YCCZ1-YCCZ2. (C) Scatter plot of YCCZ1-YCCZ3. (D) Scatter plot of YCCZ1-YCCZ4. (E) Scatter plot of YCCZ1-YCCZ5. (F) Scatter plot of YCCZ1-YCCZ6.

**Fig 3:**
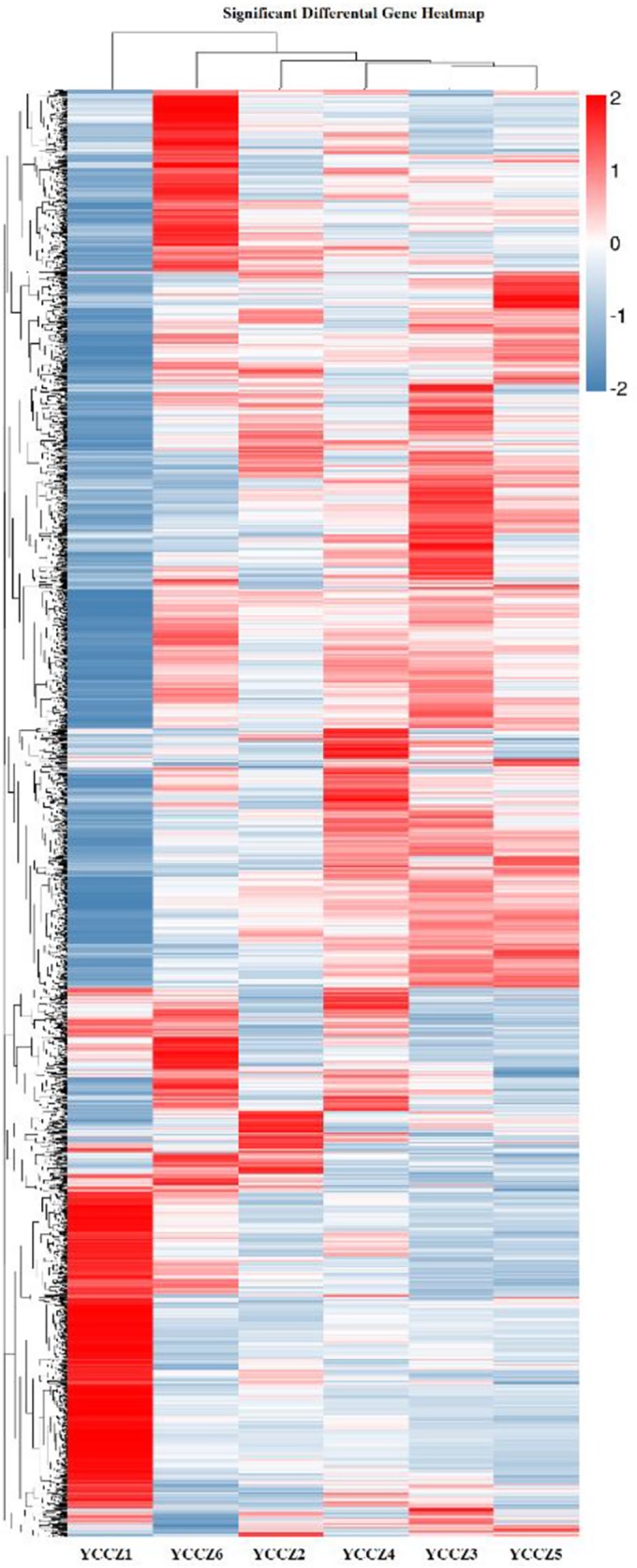
DEG-based correlation heat map (CHM) and sample cluster analysis.

Compared with YCCZ1, 1,892 DEGs were identified in YCCZ2 (1,253 up-regulated and 639 down-regulated), 2,498 in YCCZ3 (1,729 up-regulated and 769 down-regulated), 2,006 in YCCZ4 (1,511 up-regulated and 495 down-regulated), 2,273 in YCCZ5 (1,437 up-regulated and 836 down-regulated) and 2,188 in YCCZ6 (1,602 up-regulated and 586 down-regulated) (Fig 2A). The most DEGs were thus observed in the third generation, when the *C*. *militaris* fruiting body began to experience abnormal growth (Fig 1), indicating that these DEGs may play key roles in strain degeneration. In addition, a scatter plot was protracted based on DEGs analyses. Compared with YCCZ1, difference in expression levels of the DEGs in YCCZ3 and YCCZ5 was significantly higher than those in YCCZ1 and YCCZ2 (Fig 2B–Fig 2F). Furthermore, a correlation heat map (CHM) was constructed and sample cluster analysis was performed based on DEGs in different samples. The heat map of significant DEGs shows the relationship of samples YCCZ1 to YCCZ6 (Fig 3). Taken together, all results suggest that these specific DEGs may be involved in the strain degeneration progress.

### GO and KEGG pathway analyses of AS genes

**Fig 4:**
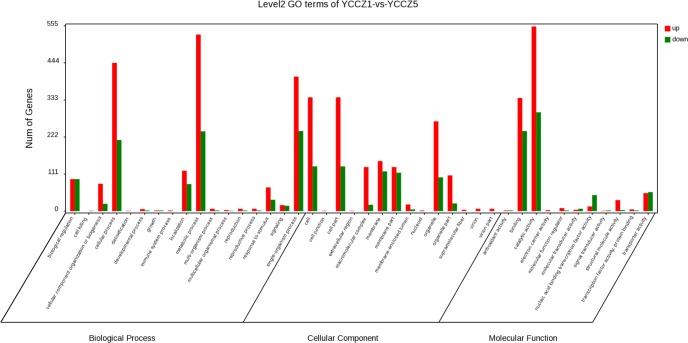
GO classification map of YCCZ1 vs. YCCZ5.

GO analysis was performed to summarize and explore the functional categories of the genes that underwent AS in different samples. GO terms were annotated for a total of 5,014 AS genes in YCCZ1, 6,230 in YCCZ2, 5,638 in YCCZ3, 6,170 in YCCZ4, 5,951 in YCCZ5 and 6,320 in YCCZ6 (S3 Fig). The Intergenic (10,863) and IR (5,222) AS events predominated, accounting for 30.8% and 14.8%, respectively, of all AS events. The number of AS genes in YCCZ2, YCCZ4 and YCCZ6 was higher than those in other samples. The proportions of enriched GO terms among AS genes were globally similar between YCCZ1 (wild-type) and YCCZ5 (degenerated) based on biological process, molecular function, and cellular component ontolopies (Fig 4). For the biological processes category, genes with AS events were predominantly enriched in GO terms that were relevant to cellular process and metabolic process. For the molecular function category, most AS genes were annotated with the term binding, followed by the term catalytic activity.

Moreover, 6,944 AS genes were annotated with KEGG pathways information (Table 2, S4 Fig). Enriched pathway analysis showed that these genes were significantly enriched in 17 pathways mainly relating to antigen processing and presentation, cell adhesion molecules, phagosome, endocytosis, and natural killer cell-mediated cytotoxicity.

**Table 2:** Enrichment KEGG pathway analysis.

list	Pathway	DEGs genes with pathway annotion (17)	All genes with pathway annotation (6944)	P value	Q value	Pathway ID
**1**	Antigen processing and presentation	6(35.29%)	81(1.17%)	0.000000	0.000001	ko04612
**2**	Cell adhesion molecules (CAMs)	6(35.29%)	159(2.29%)	0.000001	0.000016	ko04514
**3**	Phagosome	6(35.29%)	168(2.42%)	0.000002	0.000016	ko04145
**4**	Endocytosis	6(35.29%)	231(3.33)	0.000012	0.000074	ko04144
**5**	Natural killer cell mediated cytotoxicity	5(29.41%)	137(1.97%)	0.000014	0.000074	ko04650
**6**	Complement and coagulation cascades	3(17.65%)	77(1.11%)	0.000797	0.003454	ko04610
**7**	Regulation of actin cytoskeleton	3(17.65%)	216(3.11)	0.014621	0.054308	ko04810
**8**	Pancreatic secretion	2(11.76%)	104(1.5%)	0.026100	0.084825	ko04972
**9**	Oxidatie phosphorylation	2(11.76%)	141(2.03%)	0.045622	0.131797	ko00190
**10**	Vitamin digestion and absorption	1(5.88%)	24(0.35%)	0.057223	0.148780	ko04977
**11**	Nicotinate and nicotinnamide metabolism	1(5.88%)	32(0.46%)	0.075602	0.178696	ko00760
**12**	Fat digestion and absorption	1(5.88%)	39(0.56%)	0.091407	0.194538	ko04975

### Validation of DEGs by qRT-PCR

**Fig 5:**
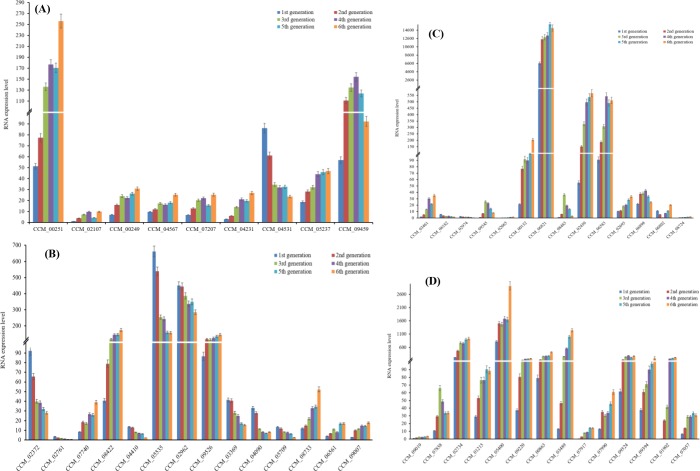
Validation of DEGs expression by qRT-PCR. (A) Genes related to DNA methylation, chromosome remodeling, mitosis, and meiosis. (B) Genes related to fruiting body formation, sexual development, light-induced brown film formation, and secondary metabolites biosynthesis. (C) Genes related to toxin biosynthesis and detoxification, and transmembrane transport. (D) Genes related to cell growth and development, cell apoptosis and autophagy, and macro molecular metabolism.

The expression of 51 DEGs was validated using qPCR, as shown in Fig 5. According to qRT-PCR, the DEG expression levels were consistent with those of obtained using RNA-Seq. Among these genes, 32 genes were up-regulated and 13 were down-regulated. Functional relative analysis shown that 15 of these genes are involved in toxin biosynthesis and detoxification (Fig 5C), 14 genes are related to carbohydrate and energy metabolism, nine genes are involved in DNA methylation and chromosome remodeling (Fig 5A), eight genes are involved in biosynthesis of secondary metabolites (Fig 5B), and six genes are involved in fruiting body formation, sexual development and light-induced brown film formation (Fig 5D). These results suggest that *C*. *militaris* strain degeneration involves genes related to toxin biosynthesis, energy metabolism, DNA methylation, and chromosome remodeling.

## Discussion

*C*. *militaris*,a model fungus belonging to the *Ascomycetes*,is an entomogenous fungus that forms a fruiting body on several types of media, such as silkworm pupa, solid rice medium, germinated soybean medium, and soybean broth. It also forms mycelia during submerged fermentation, producing several bioactive substances [19, 22].

Irreversible *C*. *militaris* strain degeneration is a common phenomenon that occurs with high frequency during the process of subculture and preservation. Such degeneration seriously affects large-scale industrialized production of *C*. *militaris*, leading to considerable economic losses [23]. There are many factors that may lead to fungal degeneration, including gene mutations, changes in mating type, DNA methylation, and fungal and viral infections [24]. Our previous work indicated that mutations in the 18S rRNA gene and mating-type (MAT) region, as well as down-regulated of *CmMAT* gene expression levels, may play important roles in *C*. *militaris* degeneration [25]. Quantitative real-time PCR analysis detected no *CmMAT1-2-1* gene expression in the degenerated strain. Expression levels of the *CmMAT1-1-1* and *CmMAT1-1-2* genes were significantly down-regulated to only 7.5% and 4.4%, respectively, that of the wild-type strain.

The *C*. *militaris* genome (147 × coverage) is predicted to have a total genome size of 32.2 Mb and over 9,684 genes [17]. In this study, 9,746 genes were detected, including 731 novel genes, which fulfilled the criterion for it being referred to as the *C*. *militaris* genome, suggesting that the genome size and gene number in *C*. *militaris* is similar to those of *Metarhizium anisopliae* (10,582 genes) [26] and *Metarhizium acridum* (9,849 genes) [27]. In addition, over 5,000 novel AS events of seven AS types were detected in each of the subcultured strains, indicating significantly higher rates of AS than in that observed in the wild-type strain. AS is an important mechanism for regulating gene expression and generating proteome diversity [28, 29]. Previous studies have shown that AS plays a decisive role in the generation of receptor diversity and regulation of growth and development [30]. Many genetic diseases have been closely linked to higher than normal rates of AS [31]. Therefore, we speculated the higher AS rates in filamentous fungi might be involved in the progression of strain degeneration.

Gene mutation [32], methylation modification [33], and DNA recombination can alter a strain’s genotype and can induce strain degeneration. In this study, we characterized and manually annotated the functions of four DEGs involved in methylation modification and in DNA recombination and repair. Methyltransferase type 11 (CCM_00251) and 6-O-methylguanine-DNA methyltransferase (CCM_02107) were significantly up-regulated in later generations and are crucial for genome stability, preventing mismatch during DNA replication and transcription [34, 35]. Two DNA repair genes, DNA excision repair protein (CCM_00249) and DNA double-strand break repair/VJ recombinationXRCC4 (CCM_04567), were also up-regulated in later generations.

Degenerated strains exhibit traits that are generally heritable, including significantly reduced sporulation, inability to form normal fruiting bodies, and significantly reduced secondary metabolites contents. Fruiting body and light-induced brown film formation areimportant characteristics that are altered in *C*. *militaris* strain degeneration [14]. Here, we analyzed the DEGs involved in fruiting body and light-induced brown film formation and found that the expression of α-1,3-mannosyltransferase*Alg3* (CCM_04231) was up-regulated while that of sexual development activator *VeA* (CCM_04531)was down-regulated. The expression of light-induced brown film formation-related genes phosducin I (CCM_05237) and phosducin II (CCM_09459) were significantly up-regulated beginning at the second generation.

Cordycepin, adenosine, polysaccharides, and ergosterol are the major bioactive constituents important in industrial cultivation [16, 36] speculated genes encoding ribonucleotide reductase, adenosine kinase, AMP deaminase, pyruvate kinase, 5’-nucleotidase among others were involved in the putative cordycepin metabolism pathway. This study showed that the expression of the genes encoding s-adenosylhomocysteine nucleosidase (CCM_02372) and oxidordeuctase (CCM_02761) was significantly down-regulated, and that of genes encoding adenine nucleotide alpha hydrolases like (CCM_07740) andadenine nucleotide alpha hydrolases like (CCM_08422) were significantly up-regulated during the degeneration progress. Ergosterol is one of the main components of the fungal cell membrane. The genes encoding dimethylallyl tryptophan synthase (CCM_04410), cytochrome P450 51 (CYP51, CCM_05535), cytochrome P450 61(CYP61, CCM_02962) were down-regulated, while that encoding lanosterol synthase (CCM_09526) was up-regulated during strain degeneration. Dimethylallyl tryptophan synthase is a key regulator of ergosterol synthesis [37, 38]. CYP51 and CYP61 are conserved members of P450 family and play important role in the biosynthesis of ergosterol [17]. qRT-PCR resultssuggested that the biosynthesis of ergosterol reduced with strain degenerations. *C*. *militaris* polysaccharidesare not only important immune regulators, but also exhibit antioxidant, antibacterial, antitumor, and antivirus activities in addition to other biological activities [1]. Several genes are related to the metabolism of *C*. *militaris* polysaccharide, including1,4-α-glucan branching enzyme(CCM_03369), α-N-acetylglucosaminidase (CCM_04090), mannan endo-1,6-α-mannosidase (CCM_05709), sorbitol dehydrogenase (CCM_06561), mannosidase MsdS (CCM_08733), and sorbitol utilization protein (CCM_09007)[39].

Toxic substances such as mycotoxins, active oxygen and other nonnutritional xenobiotic compounds, naturally exist in the environment and can be harmful to organisms [40]. Some scholars have speculated that accumulation of toxin and active oxygen molecules may cause fungal strain degeneration [14]. Here, the expression of several genes involved in detoxification was up-regulated during strain degeneration, including that encoding streptothricin-acetyltransferase (CCM_00152), which degrades streptothricin [41]; gamma-glutamyltranspeptidase (CCM_02065), which reduces glutathione [41]; MFS multidrug transporter (CCM_02974), which confers resistance to antibiotics [42]; glutathione S-transferase (CCM_03461), which processes pesticides, heavy metals, fluoride and eliminating reactive oxygen species (ROS) [43,44]; 30 kDa heat shock protein (CCM_06821), which is involved in stress response, signal transduction, and xenobiotic compounds metabolism [45]; and alcohol dehydrogenase (CCM_09345), which ameliorates the harmful effects of high concentration of ethanol [46]. However, expression of the gene encoding heavy metal tolerance protein (CCM_06182), which binds heavy metals ions, was down-regulated during strain degeneration [47]. In addition, levels of non-nutritional xenobiotic compounds increased during strain degeneration induced by subculture.

In contrast, expression of genes related to the metabolism of carbohydrate, lipid, protein, amino acid, nucleic acid, and nucleotide was significantly up-regulated during strain degeneration. These included the genes encoding mitochondrial hypoxia responsive protein (CCM_05400), mitochondrial co-chaperone GrpE (CCM_00863), glycoside hydrolase, family 2 (CCM_09220), trypsin-like serine protease (CCM_03489), metalloprotease 1 (CCM_07917), acetate transporter (CCM_07990), SGT1 and CS protein (CCM_09524), formyltetrahydrofolate deformylase (CCM_09394), nucleoside triphosphate hydrolases like (CCM_01902), and uracil phosphoribosyltransferase (CCM_07057).

The subcultured *C*. *militari*s strains degenerated beginning at the third generation, with incomplete fruiting body growth beginning at the fourth generation. Transcriptome analysis on the progress of *C*. *militaris* subculture degeneration showed that over 9,015 unigenes and 731 new genes were identified, and 35,323 alternative splicing (AS) events were detected. Compared with the first generation, the third generation (degenerated strain) included 2,498 differentially expressed genes (DEGs) including 1,729 up-regulated and 769 down-regulated genes. Validation of RNA-seq by qRT-PCR showed that the expression patterns of 51 DEGs were in accordance with the transcriptome data. In summary, our results indicated the mechanism of *C*. *militaris* strain degeneration is associated with gene involved in toxin biosynthesis, energy metabolism, DNA methylation, and chromosome remodeling.

## Supporting information

S1 Fig Classification of clean reads. (TIF)

S2 Fig Distribution of gene coverage. (TIF)

S3 Fig Alternative splicing (AS) distribution of the six samples. (TIF)

S4 Fig KEGG pathway enrichment analysis. (TIF)

S1 Table Primers used for qRT-PCR. (TIF)

S2 Table Sequence numbers of the clean data. (TIF)

S3 Table Sequence numbers of the read data. (TIF)

## Abbreviations

C. militaris: *Cordyceps militari*s
AS: alternative splicing
DEGs: differentially expressed genes
GO: Gene Ontology
KEGG: Kyoto Encyclopedia of Genes and Genomes
qRT-PCR: quantitative real-time PCR
YCC: the strain of *C*. *militaris* used in this study
PDA: potato dextrose agar
CmGAPDH: *C*. *militaris* housekeeping gene, glyceraldehyde-phosphate dehydrogenase
CHM: correlation heat map
MAT: mating-type
CYP51: cytochrome P450 51
CYP61: cytochrome P450 61
ROS: reactive oxygen species
