# Supplementary figures and images for: Transcriptome-wide analysis reveals the progress of Cordyceps militaris subculture degeneration
Source: PLoS One. 2017 Oct 26;12(10):e0186279. doi: 10.1371/journal.pone.0186279 (PMC5657973; doi:10.1371/journal.pone.0186279)

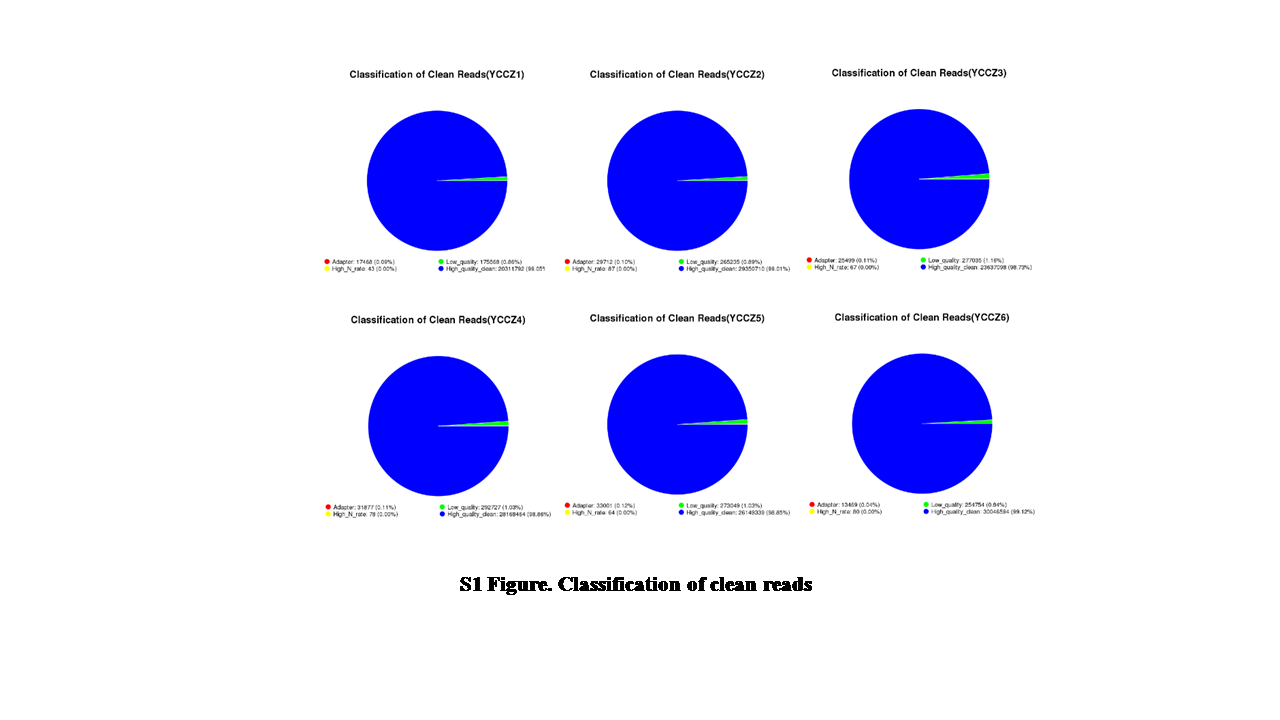

Supplement: S1 Fig — (TIF) [file pone.0186279.s001.tif]

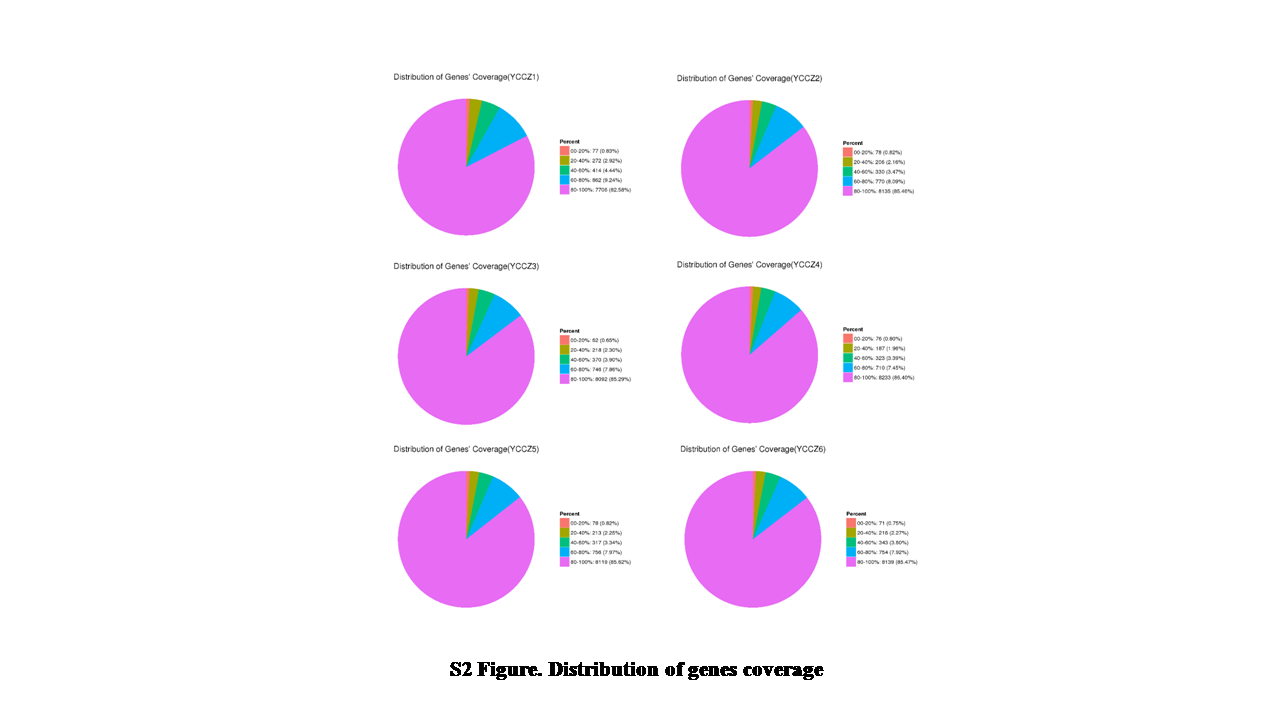

Supplement: S2 Fig — (TIF) [file pone.0186279.s002.tif]

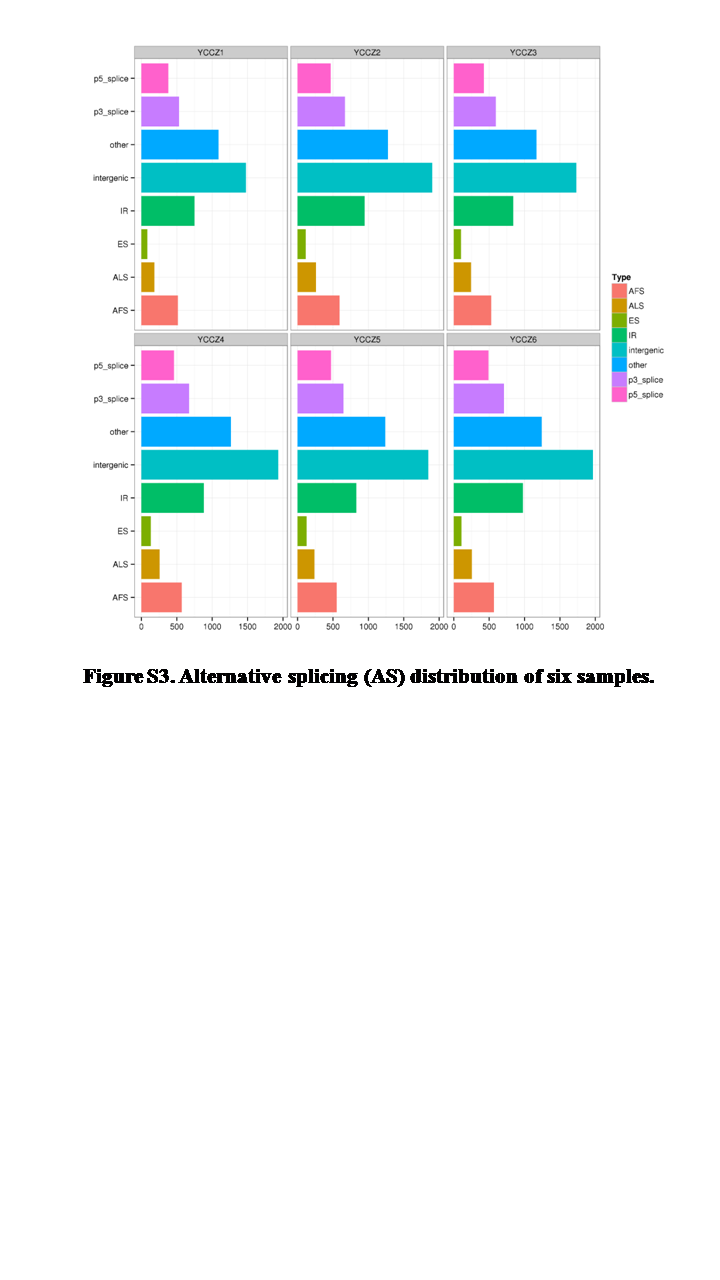

Supplement: S3 Fig — (TIF) [file pone.0186279.s003.tif]

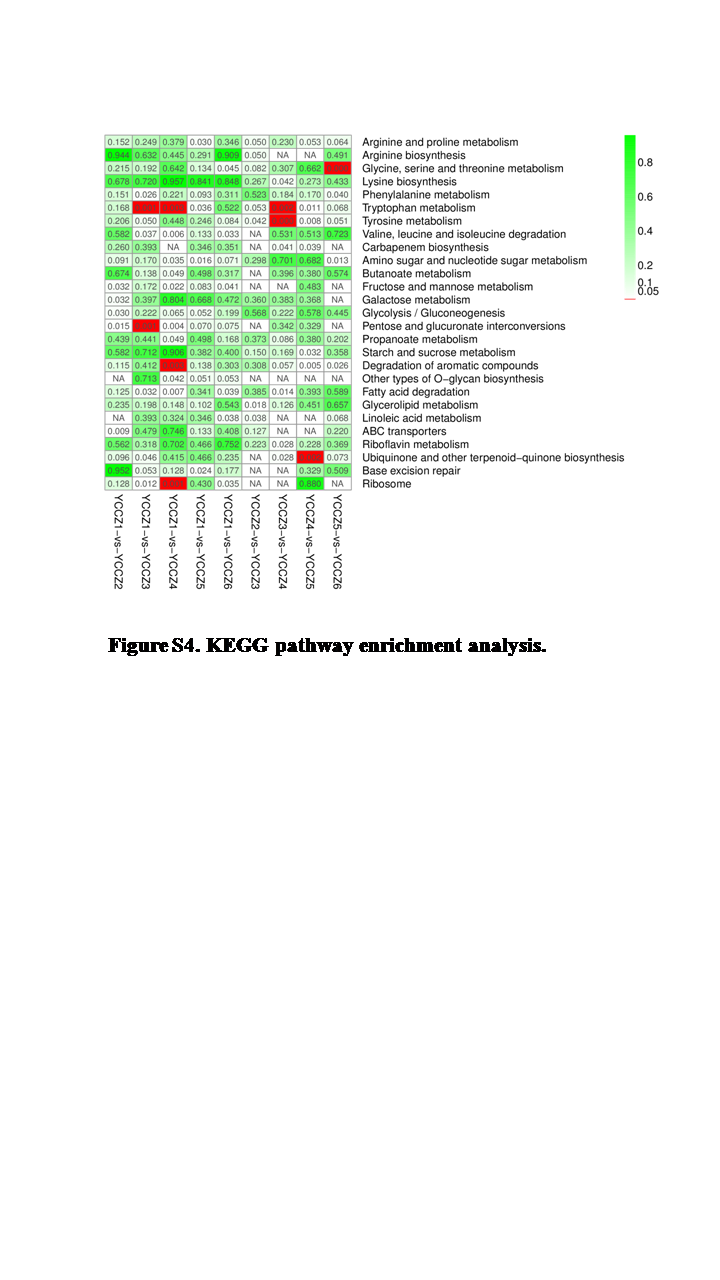

Supplement: S4 Fig — (TIF) [file pone.0186279.s004.tif]

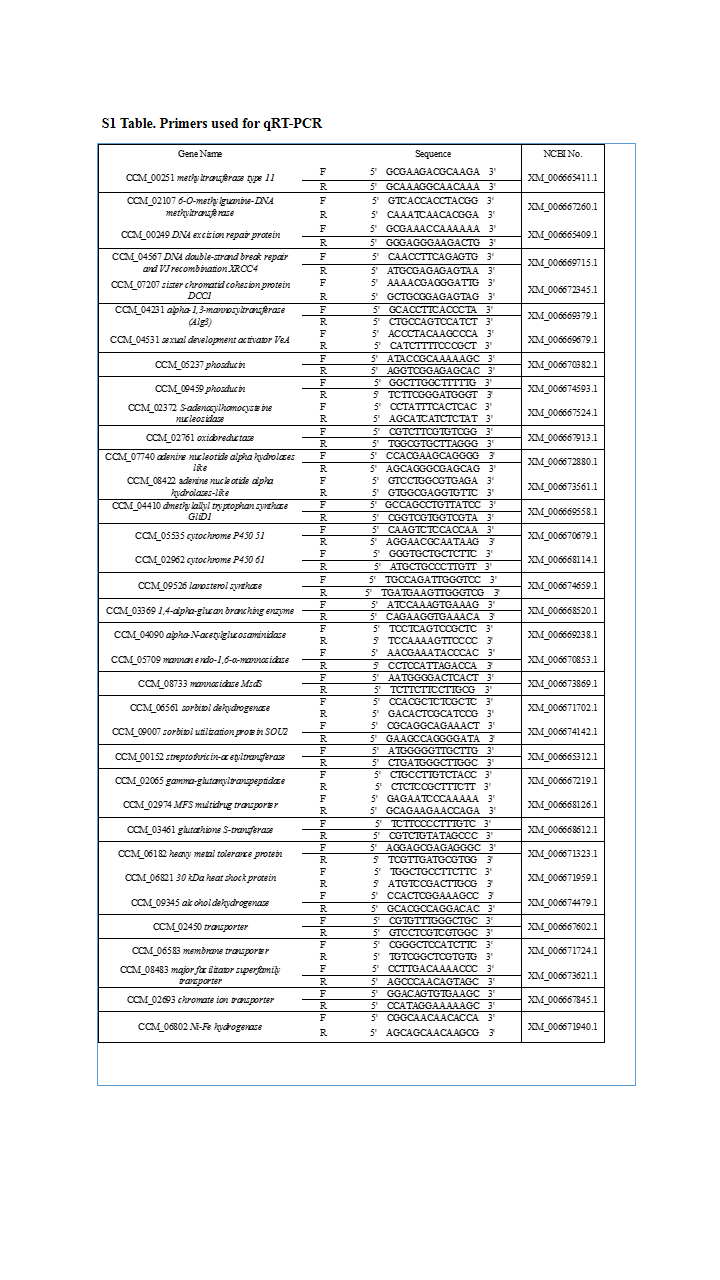

Supplement: S1 Table — (TIF) [file pone.0186279.s005.tif]

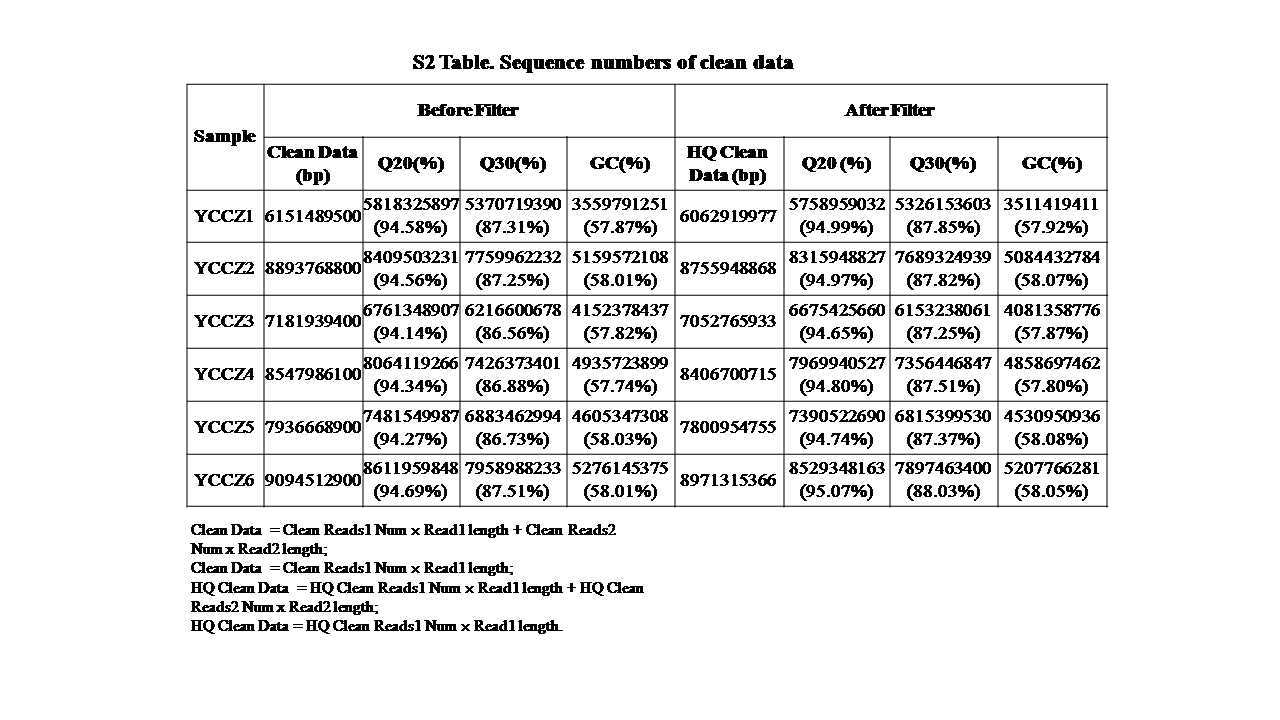

Supplement: S2 Table — (TIF) [file pone.0186279.s006.tif]

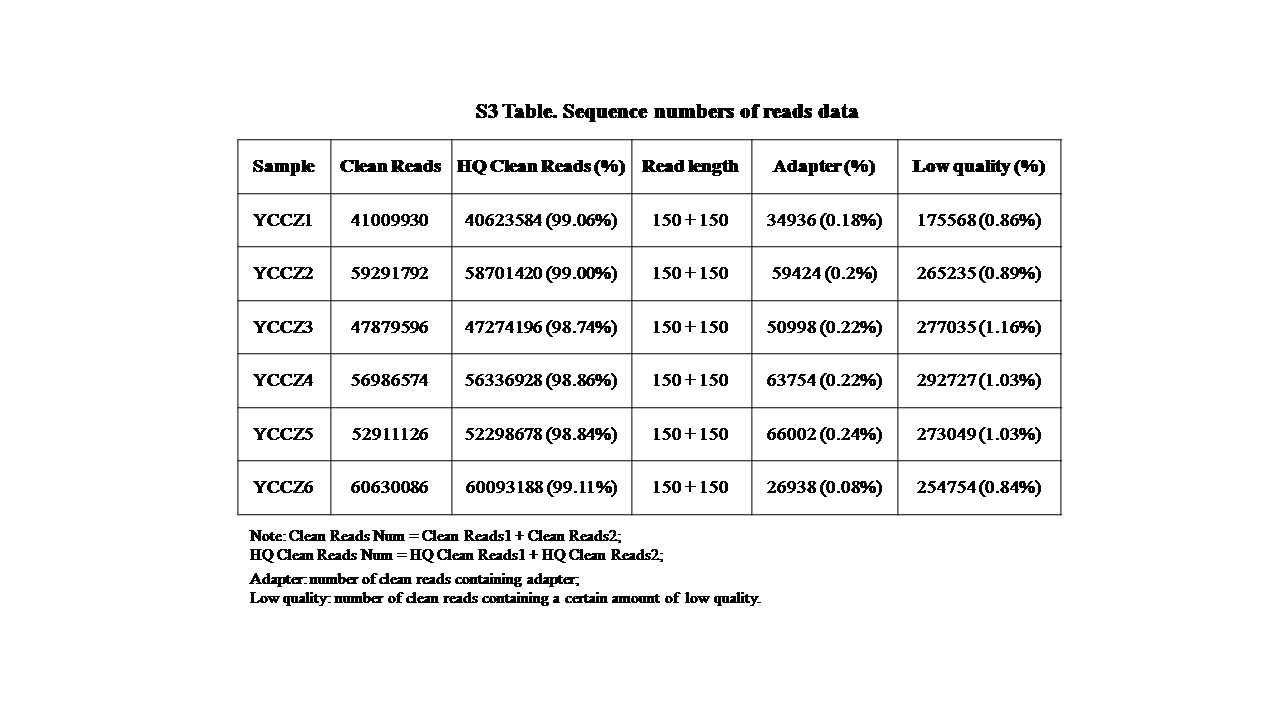

Supplement: S3 Table — (TIF) [file pone.0186279.s007.tif]
